# Early periodontal wound healing after chlorhexidine rinsing: a randomized clinical trial

**DOI:** 10.1007/s00784-024-05643-0

**Published:** 2024-06-04

**Authors:** Filippo Graziani, Rossana Izzetti, Marina Perić, Urška Marhl, Marco Nisi, Stefano Gennai

**Affiliations:** 1 Department of Surgical, Medical and Molecular Pathology and Critical Care Medicine, University of Pisa, Pisa, Italy; 2 University of Split School of Medicine, Šoltanska 2, 21000 Split, Croatia; 3 Community Healthcare Centre Dr. Adolf Drolc Maribor, Ulica Talcev 9, 2000 Maribor, Slovenia

**Keywords:** Hyaluronic acid, Chlorhexidine, Periodontal surgery, Wound healing

## Abstract

**Objectives:**

This single-center randomized, parallel design, clinical trial with a 2-week follow-up involved patients affected by periodontitis undergoing periodontal surgery. The aim was to evaluate periodontal surgical wound healing with the use of chlorhexidine-based mouth rinses versus an untreated control group.

**Materials and methods:**

Periodontal surgery was performed following a standardized protocol. Patients were randomly prescribed i) chlorhexidine (CHX) + anti-discoloration system (ADS) + hyaluronic acid (HA), ii) CHX + ADS or iii) no treatment (control group). Plaque score, gingival inflammation, and Early Healing Index (EHI), assessing the degree of wound closure and the presence of fibrin and necrosis, were evaluated at 3, 7 and 14 days after surgery.

**Results:**

In total, 33 patients were enrolled. Patients were comparable at baseline for all measured clinical parameters. At 3-days wound healing was significantly improved in all patients treated with CHX + ADS-based mouth rinses with a lower EHI score at the interdental papillae compared with control group (*p* < 0.01). CHX + ADS + HA group presented improved healing across all time points in terms of EHI, plaque containment, and gingival inflammation when compared to control group (*p* < 0.01).

**Conclusions:**

The usage of CHX-ADS following periodontal surgery improved early wound healing, reduced plaque accumulation and gingival inflammation. During the early post-operative period the adjunct of HA further improved soft tissue closure.

**Clinical relevance:**

This study aims at evaluating the response of gingival tissues to mouth rinsing with chlorhexidine and anti-discoloration system (CHX + ADS) or CHX + ADS + hyaluronic acid (CHX + ADS + HA) versus no rinse in terms of healing of the periodontal surgical wound. CHX + ADS mouth rinses enhanced early soft tissue closure after periodontal surgery and contributed to the reduction in plaque accumulation and gingival inflammation. The adjunct of HA may be beneficial especially in the early post-operative period. CHX + ADS administration following periodontal surgery may improve soft tissue healing in the first two post-operative weeks.

**Supplementary Information:**

The online version contains supplementary material available at 10.1007/s00784-024-05643-0.

## Introduction

Primary wound closure is one of the aims of periodontal surgery, with the first postoperative week being critical for the maintenance of wound stability [34, 50]. In periodontal surgical wounds, the healing process begins in the first 12–24 h post-surgery, when the initial migration of the keratinocytes begins to restore tissue continuity and protects the wound from microbiological and mechanical impact [51]. Several factors are recognized to concur to post-operative course and the obtaining of primary wound closure. The prevention of biofilm formation and the reduction of infective complications on surgical wound, as well as wound stability, are important factors for the success of the procedure [41, 48]. However, post-operative oral hygiene instructions are often insufficient in providing adequate plaque control and preventing bacterial colonization of the surgical site [13].

In the early post-operative period, chemical agents can be used as an adjunct to mechanical plaque removal to control oral biofilm formation [29, 37, 40, 49]. Chlorhexidine (CHX) is the agent of choice due to its effectiveness in reducing plaque formation by the 30–80%, and its use is well-established [6, 8–10, 22, 25, 27, 38]. The combination of CHX with hyaluronic acid has been reported to improve periodontal parameters, reduce inflammation, and support wound healing process [1, 7, 18, 32, 42]. In fact, hyaluronic acid has been claimed to have numerous beneficial properties on wound healing due to its bacteriostatic [11] fungistatic [23], anti-inflammatory [39], anti-oedematous [15], osteo-inductive [39] and pro-angiogenetic properties [17]. During the healing process of oral wounds, hyaluronic acid promotes neo-angiogenesis and fibroblasts proliferation, along with collagen maturation and extra-cellular matrix remodelling [35]. Clinical and histological evidence also supports the usage of hyaluronic acid in the early healing period following periodontal surgery, accounting for an overall improvement in clinical wound healing response [33].

Therefore, the aim of this study was to evaluate periodontal wound healing in patients treated with chlorhexidine-based mouth rinse with or without hyaluronic acid after periodontal surgery in an in vivo wound healing model for periodontal surgery.

## Materials and methods

### Patient selection

This study was a single-center randomized, parallel design, clinical trial with a 2-week follow-up duration. The study protocol was approved by the local ethics committee of the University Hospital of Pisa (CEAVNO, protocol no. 55143) and registered within a clinical trial database (ClinicalTrials.gov, NCT04345744). The study was conducted according to the principles of the Declaration of Helsinki on experimentation involving human subjects. All patients signed an informed consent form to participate in the study.

Patients treated for their periodontitis and eligible for periodontal surgery were enrolled at the Sub-Unit of Periodontology, Halitosis and Periodontal Medicine, University Hospital of Pisa (Italy).

Eligibility criteria were: i) males or females of age range between 18 and 70 years, ii) systemically healthy patients, iii) Stage III grade B-C periodontitis), iv) mixed defects presenting a residual PPD ≥ 6 mm following Step I of periodontal treatment without involvement of furcations; v) patients willing to give informed consent, and vi) compliance to the study follow-up.

Exclusion criteria were: i) age < 18 years or > 70 years, ii) pregnancy or breast-feeding, iii) therapy with oral contraceptives, iv) indication to antibiotic therapy prior to surgical treatment, vi) systemic diseases (including cardiovascular, pulmonary, cerebral, and metabolic diseases), vii) usage of chlorhexidine mouth rinses in the 6 months prior to the enrolment, and viii) heavy smoking habit (> 20 cigarettes per day, and/or pipe or cigar smoking).

### Study design and in-vivo model of wound healing

Patients who were previously treated non-surgically for their periodontitis were re-assessed and invited to participate in the study if the presence of at least a residual site of ≥ 6mm was detected. All participants, after receiving thorough and detailed information about the study, gave informed written consent. After inclusion, data on medical and dental history were recorded for each study participant.

Papilla preservation flap was performed by a single operator. The flaps used were mainly Minimally Invasive Surgical Technique (MIST) [14] and Single Flap Approach (SFA) [43]. No attempts to perform anatomical changes were performed, thus nor resective nor reconstructive approaches were involved in the surgical interventions. No regenerative materials were applied surgically. Briefly, the papilla associated with the defect was exclusively involved in the incision with a careful elevation of the buccal and palatal components to a very limited extent up to 1–2 mm of alveolar bone crest. A releasing incision (experimental incision) was always performed in the keratinized mucosa at the border of the surgical area and did not involve the papillary area to avoid any effect on the healing process of the treated defect. The wound healing in-vivo model comprised two incisions: the one at the base of the papilla and the releasing incision. Since no regenerative procedures were performed, the papillary area was sutured with single interrupted interdental suture, providing a close interdental adaptation between the buccal and lingual flaps with equal tension on both sides. Then, the stability of the flap was tested. If the flap was stable, no suture was applied in the releasing incision, otherwise single interrupted sutures were given to further stabilize the flap. The type of defect was deemed uninfluential to the assessment of wound healing being the surgical approach conservative. In fact, the degree of wound healing was assessed on tissues repositioned to achieve primary intention wound closure.

After surgery, participants were randomly allocated to one of three experimental groups. Randomization was performed through a computer‐generated table and concealed to the clinical examiner and statistician with sequentially numbered sealed opaque envelopes that were opened on the day of allocation by a clinical staff member.

The experimental groups corresponding to mouth rinse prescription were:

- CHX + ADS + HA group: administration of 0.2% chlorhexidine + anti-discoloration system + 0.2% hyaluronic acid mouth rinse (Curasept S.p.A., Saronno, Italy);
- CHX + ADS group: administration of 0.2% chlorhexidine + anti-discoloration system mouth rinse (Curasept S.p.A., Saronno, Italy);
- CTRL group: no mouth rinse was prescribed.

Patients were blind to treatment as they received a dark coded mouth rinse bottle. The mouth rinse protocol consisted of a 10 ml-rinse for 60 s twice-a-day (every 12 h) for 14 days.

### Post-surgical protocol

Mouth rinsing was clearly explained after surgery and patients were advised to begin the same day of the surgical intervention. Patients were instructed in oral hygiene manoeuvres and prescribed the use of a post-operative (post-op) toothbrush with ultra-soft bristles, to be used according to the Bass brushing technique. Patients were instructed to refrain from using interdental devices in the surgical sites during the follow-up period.

**Fig. 1:**
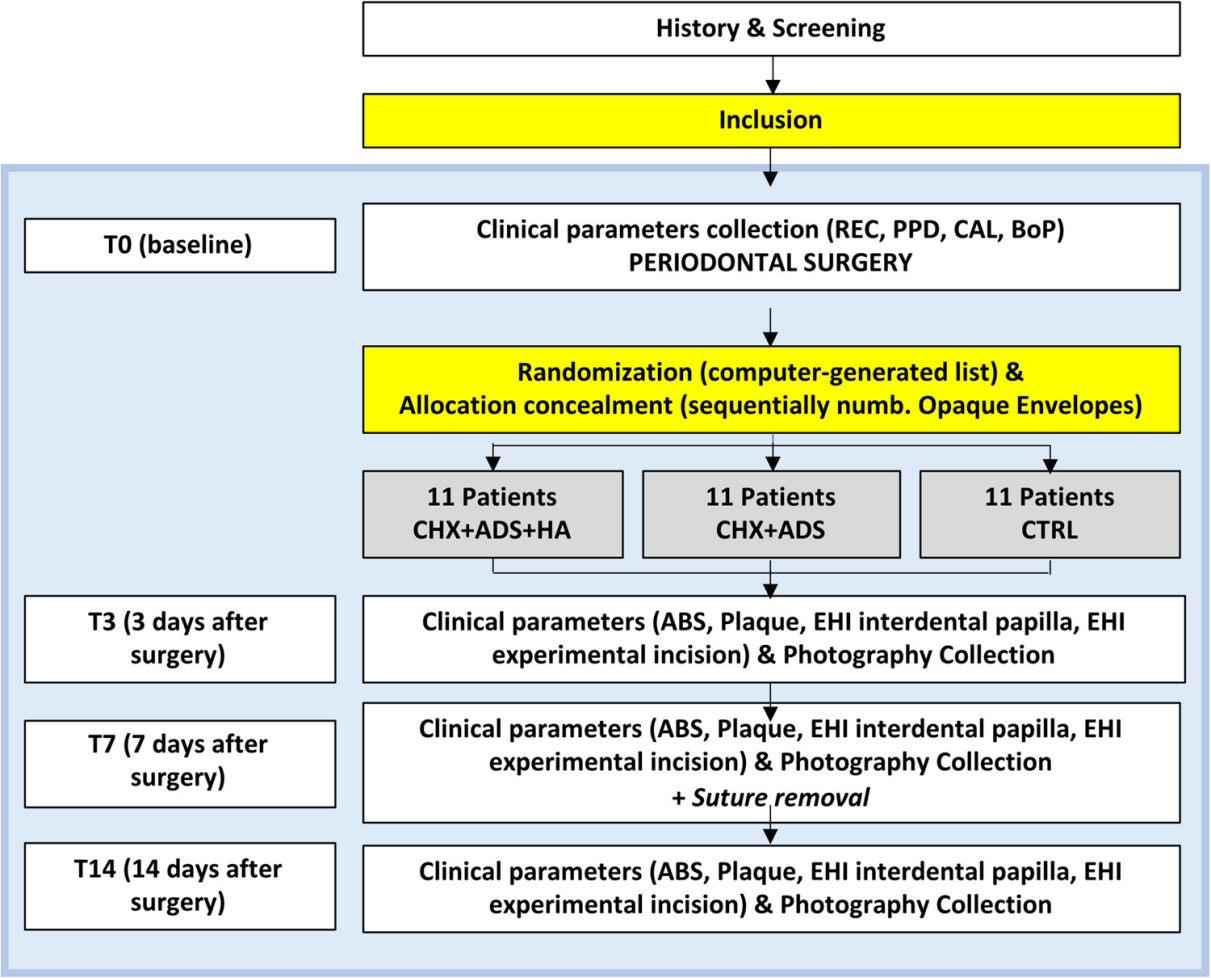
Study flowchart

Enrolled participants were examined at 3, 7 and 14 days after surgery to assess the degree of wound healing. Clinical pictures of the surgical area were also taken. Study flowchart is depicted in Fig. 1.

### Clinical parameters

Full-mouth plaque scores were assessed dichotomously, evaluating the presence/absence of plaque at six sites per tooth [2, 30]. Angulated Bleeding Score (ABS) was calculated with the probe running along the marginal gingiva and held at an angle of approximately 60° to the longitudinal axis of the tooth. Score 0 corresponded to absence of bleeding, and Score 1 to bleeding upon probe stimulation [44].

### Wound healing assessment

The degree of surgical healing was clinically assessed using the Early Wound Healing Index (Wachtel classification; EHI), proposed by Wachtel and collaborators [47]. Briefly, the degree of wound healing was classified as follows:

- Score 1: complete wound healing: absence of fibrin line in the interproximal area
- Score 2: complete wound healing: presence of a thin fibrin line in the interproximal area
- Score 3: complete wound healing: presence of fibrin clot in the interproximal area
- Score 4: incomplete wound healing: presence of partial necrosis of the interproximal area
- Score 5: incomplete wound healing: total necrosis of the interproximal area.

The EHI score was clinically evaluated both at the level of the experimental incision (EHI experimental incision) and at the level of the interdental papillae involved (EHI interdental papillae). Both parameters were collected at T3, T7 and T14, by two different operators. The degree of wound healing was further classified dichotomously at all timepoints as complete wound healing (scores 1–3) and incomplete healing (scores 4–5). The presence of dehiscence, defined as partial or total separation of previously approximated wound edges due to a failure of proper wound healing, was also assessed at the level of the primary incision.

### Re-assessment evaluation

**Fig. 2:**
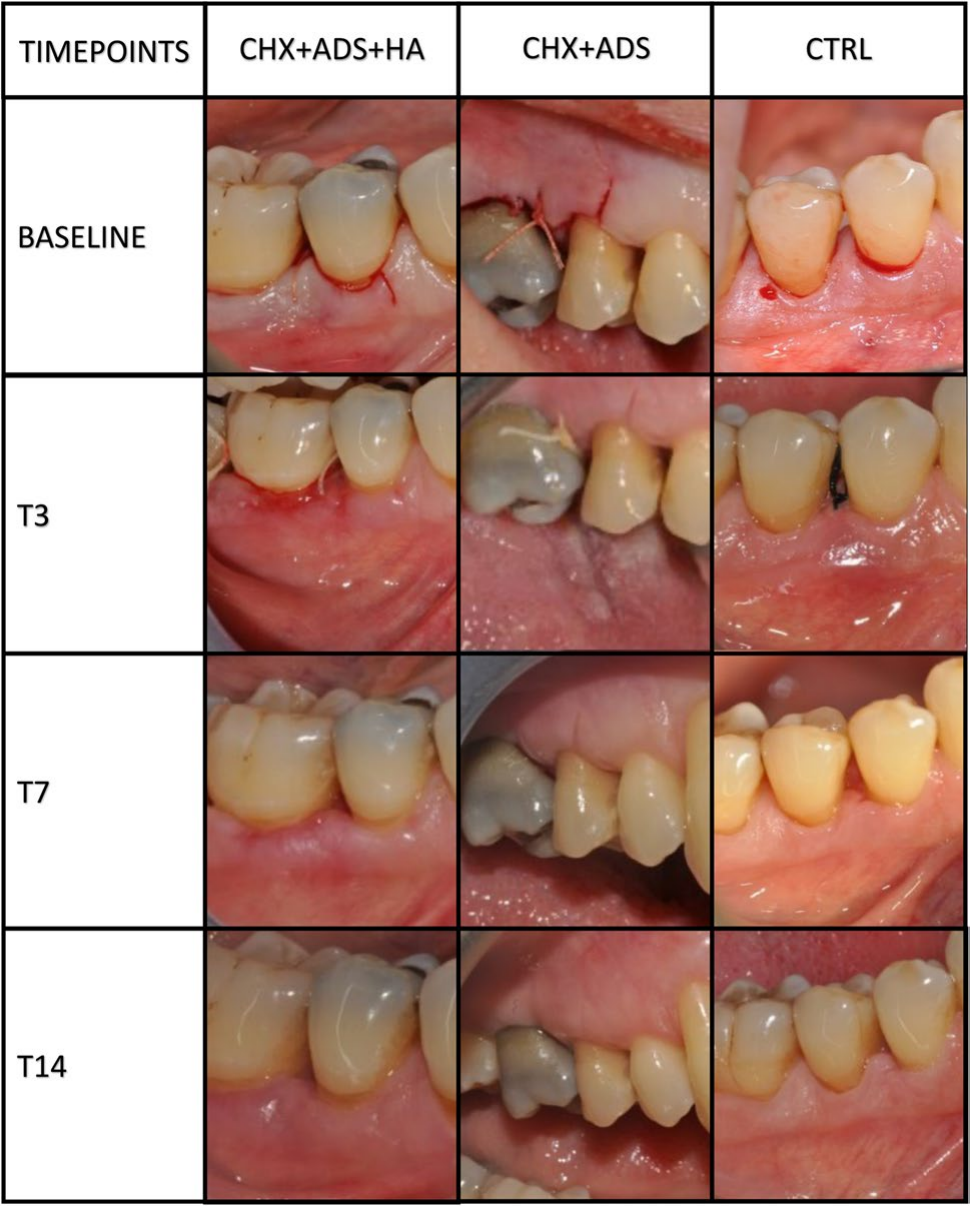
Clinical photographs of the wound healing progression in the three study groups

At T3, T7, and T14, the EHI score was clinically evaluated at the level of the experimental incision and the interdental papilla, and PPD, Rec, and PI were recorded for each tooth in the surgical area. Supragingival polish was performed at each timepoint (T3, T7 and T14) (Fig. 2).

At T3, the first follow-up examination was performed. Periodontal scores were registered and EHI evaluated.

At T7 (second follow-up examination), clinical parameters registration and EHI assessment were repeated, and suture was removed.

At T14 (third follow-up examination), the last registration of periodontal and EHI scores were performed. This timepoint corresponded to the end of the study.

### Calibration

For the assessment of wound healing at the level of the papilla and at the level of the experimental incision, intra-examiner calibration was performed after training with an experienced trialist on 10 calibration rounds for clinical EHI scores, until a Cohen kappa score > 0.8 was reached.

### Sample size calculation

The aim was to evaluate wound healing in patients treated with chlorhexidine-based mouth rinse after periodontal surgery. Since the change in EHI was the primary outcome measure, an estimate of the sample size was made using the following assumptions, based on a previous study by [47] on periodontal surgical wound healing [47]: significance level α = 0.05, power = 0.9 and difference in proportion = 0. These hypotheses required a sample size of at least 11 subjects (per group) to obtain valid and reliable results, capable of detecting a significant difference.

### Statistical analysis

All data are presented as mean and standard deviation unless otherwise specified. Patient was a statistical unit. Analysis of variance (ANOVA) for repeated measures was employed to evaluate changes within groups from T0 to T14. Friedman test was used for data that did not follow the Gaussian distribution. Differences between groups were evaluated using Student t-test for independent data. In cases of not normally distributed data, data logging prior to statistical analysis and non-parametric Mann–Whitney U-test were applied. Bonferroni post hoc corrections were adopted. All analyses were performed with SPSS version 26.0 (SPSS Inc. Chicago, IL, USA). Statistical significance was set at *p* < 0.05.

## Results

### Baseline characteristics

Thirty-three patients (15 females and 18 males, 11 per experimental group) were included in the study. All patients completed the study and contributed to the final analysis. No dropouts were recorded. All the recruited patients were Caucasians. Mean age was 51.13 years (SD 9.92 years, age range 30–67 years). Patients were comparable for all the assessed demographic and clinical parameters at baseline. Defects were mainly suprabony or supra/intrabony defects with no differences among groups. Healing progression at the different study timepoints is depicted in Fig. 2.

### Clinical parameters at T3

EHI scores were significantly lower for the CHX + ADS + HA group at the level of the experimental incision compared to CTRL group (*p* < 0.05). No other significant differences among groups were noted. At the interdental papilla, both the groups receiving CHX performed significantly better in terms of wound healing compared to CTRL group (*p* < 0.01). In particular, CHX + ADS + HA showed lower EHI scores compared to CHX + ADS (*p* < 0.05). The EHI scores ranged between 1 and 2 in the CHX + ADS + HA group, between 1 and 3 in the CHX + ADS group and between 1 and 4 in the CTRL group.

Complete healing was observed in 72.73% of the CHX + ADS + HA group, accounting for a significantly higher proportion with respect to CHX + ADS (36.37%, *p* < 0.05) and CTRL (27.27%, *p* < 0.05) groups.

A significant difference in terms of presence of dehiscence was noted in the CTRL group compared to the CHX + ADS + HA group (*p* < 0.05), while no differences were found between CHX + ADS and CHX + ADS + HA and CTRL group, respectively.

ABS (%) was significantly lower in the CHX + ADS + HA group (*p* < 0.01 when compared to both CHX + ADS and CTRL groups). The CHX treatment groups showed significantly lower levels of plaque accumulation compared to CTRL group (*p* < 0.01 for CHX + ADS + HA and *p* < 0.05 for CHX + ADS).

### Clinical parameters at T7

Statistically significant difference was observed between the EHI scores at the level of the experimental incision in the CHX + ADS + HA group versus CTRL group (*p* < 0.01), and between the CHX + ADS group versus CTRL group (*p* < 0.05). Mean EHI scores at the level of the interdental papillae statistically differed when comparing CHX + ADS + HA with CTRL group (*p* < 0.01) and CHX + ADS with CTRL group (*p* < 0.01). EHI scores ranged between 1–2 in the CHX + ADS + HA group, while CHX + ADS and CTRL groups showed scores between 1 and 3.

Complete healing was encountered in 90.91% of patients receiving CHX + ADS + HA. The proportion of patients showing complete healing was significantly lower in the CHX + ADS (54.55%, *p* < 0.05) and CTRL (27.27%, *p* < 0.05) groups compared to CHX + ADS + HA group. The comparison between CHX + ADS and CTRL group revealed a significantly higher number of patients with complete healing in the CHX + ADS group (*p* < 0.05).

At T7, ABS (%) was significantly lower in the CHX + ADS + HA group compared to CHX + ADS (*p* < 0.01) and CTRL group (*p* < 0.01) (Table 1). Significantly lower levels of plaque accumulation were observed in the CHX + ADS + HA group compared to the CTRL group (*p* < 0.01).

**Table 1:** Early Healing Index (EHI) and clinical parameters at T3, T7 and T14

	Group				*p* value inter-group		
	CHX + ADS + HA	CHX + ADS	CTRL	*p*-value before pairwise evaluation	CHX + ADS + HA vs. CHX + ADS	CHX + ADS + HA vs. CTRL	CHX + ADS vs. CTRL
Variables at T3							
EHI -experimental	1.27 (0.47) [1–2]	1.81 (0.87) [1–3]	2.36 (0.92) [1–4]	< 0.05	NS	< 0.01	NS
EHI—interdental	1.15 (0.26) [1–2]	1.67 (0.45) [1–3]	2.45 (0.60) [1–4]	< 0.05	< 0.05	< 0.01	< 0.01
ABS, %	5.55 (8.71)	22.40 (12.30)	25.09 (15.20)	< 0.01	< 0.01	< 0.01	NS
Plaque, %	13.00 (11.02)	24.50 (17.78)	46.55 (25.46)	< 0.05	NS	< 0.01	< 0.05
Complete healing (no. of patients)	8	4	3	< 0.05	< 0.05	< 0.05	NS
Variables at T7							
EHI -experimental	1.10 (0.30) [1–2]	1.46 (0.69) [1–3]	2.18 (0.87) [1–3]	< 0.05	NS	< 0.01	< 0.05
EHI—interdental	1.09 (0.18) [1–2]	1.46 (0.34) [1–3]	2.21 (0.73) [1–3]	< 0.05	NS	< 0.01	< 0.01
ABS, %	3.36 (4.86)	29.73 (20.50)	31.36 (22.62)	< 0.01	< 0.01	< 0.01	NS
Plaque, %	11.45 (10.34)	22.45 (16.31)	40.18 (30.06)	< 0.05	NS	< 0.01	NS
Complete healing (no. of patients)	10	6	3	< 0.05	< 0.05	< 0.05	< 0.05
Variable at T14							
EHI -experimental	1.09 (0.17) [1–2]	1.45 (0.52) [1–2]	1.60 (0.51) [1–2]	< 0.05	NS	< 0.05	NS
EHI—interdental	1.08 (0.18) [1–2]	1.25 (0.25) [1–2]	1.50 (0.48) [1–2]	< 0.05	NS	< 0.01	< 0.05
ABS, %	2.18 (3.74)	20.30 (19.60)	34.20 (30.40)	< 0.05	NS	< 0.01	NS
Plaque, %	9.81 (10.36)	21.00 (13.24)	33.00 (28.03)	< 0.05	NS	< 0.01	NS
Complete healing (no. of patients)	10	7	4	< 0.05	< 0.05	< 0.05	< 0.05

All variables are expressed as mean (SD). For EHI experimental and interdental, minimum and maximum scores at each timepoint are reported in square brackets. Mann-Whitney test was employed to compare EHI scores between groups. ANOVA was used for the assessment of ABS and Plaque variations between timepoints

Abbreviations. *ABS* angulated bleeding score, *ADS* anti-discoloration system, *CHX* chlorhexidine, *CTRL* control, *EHI* early healing index, *HA* hyaluronic acid, *NS* non-significant

Dehiscence occurrence was significantly higher in the CTRL group compared to the CHX + ADS + HA group (*p* < 0.05), while no differences were found between CHX + ADS and CHX + ADS + HA and CTRL group, respectively.

### Clinical parameters T14

The EHI scores at the level of the experimental incision showed that the CHX + ADS + HA healed significantly better than CTRL group (*p* < 0.05), while no differences were registered between CHX + ADS + HA and CHX + ADS and between CHX + ADS and CTRL group. At the interdental papillae, both CHX groups presented lower EHI scores compared to CTRL group (*p* < 0.01 for the CHX + ADS + HA group and *p* < 0.05 for the CHX + ADS group). At T14 EHI scores ranged between 1 and 2 in all groups. Complete healing occurred in 90.91% of patients in the CHX + ADS + HA group, in 36.36% of the CTRL group and in 63.64% of the CHX + ADS group. Statistically significant differences in complete healing were observed when comparing CHX + ADS + HA with CHX + ADS (*p* < 0.05) and CTRL group (*p* < 0.05), and when comparing CHX + ADS and CTRL group (*p* < 0.05). No differences between groups in terms of presence of dehiscence were registered at this timepoint.

ABS (%) and plaque accumulation (%) were significantly lower in the group receiving CHX + ADS + HA with respect to CTRL group. No differences were registered when setting comparison with the CHX + ADS group. The progression of wound healing assessed at the different timepoints is reported in Table 1 and 2.

**Table 2:** Presence of dehiscence in the three groups at the study timepoints. Statistical analysis was performed with ANOVA

	Group	CHX + ADS	CHX + ADS + HA	CONTROL	CHX + ADS vs. CHX + ADS + HA	CHX + ADS vs. CONTROL	CHX + ADS + HA vs. CONTROL
T3	Not dehiscent	27.27	50.00	18.18	NS	NS	< 0.5
	Dehiscent	72.73	50.00	81.82	NS	NS	< 0.5
T7	Not dehiscent	36.36	61.11	27.27	NS	NS	< 0.5
	Dehiscent	63.64	38.89	72.73	NS	NS	< 0.5
T14	Not dehiscent	36.36	61.11	36.36	NS	NS	NS
	Dehiscent	63.64	38.89	63.64	NS	NS	NS

*ADS* anti-discoloration system, *CHX* chlorhexidine, *CTRL* control, *HA* hyaluronic acid, *NS* non-significant

### Intra-group analysis

Intra-group variations did not highlight significant changes in terms of average mean values of ABS (%). In the CTRL group, plaque accumulation was significantly lower between T3 and T14 (*p* < 0.05). The EHI scores at the experimental incision decreased significantly from T3 to T7 (*p* < 0.05) and from T3 to T14 (*p* < 0.05) in the CHX + ADS group. In the CTRL group, statistically significant changes were observed between all timepoints (T3 versus T7, *p* < 0.05; T7 versus T14, *p* < 0.05; T3 versus T14, *p* < 0.01). The mean EHI at the level of the interdental papillae showed significant reduction across all time points in the CTRL group (*p* < 0.05) (Table 3).

**Table 3:** Intra-group analysis. Statistical analysis was performed with ANOVA

Variable	Group	*p* value intra-group		
		T3 vs. T7	T3 vs. T14	T7 vs. T14
EHI experimental	CHX + ADS + HA	NS	NS	NS
	CHX + ADS	< 0.05	< 0.05	NS
	CTRL	< 0.05	< 0.01	< 0.05
EHI interdental	CHX + ADS + HA	NS	NS	NS
	CHX + ADS	NS	NS	NS
	CTRL	< 0.05	< 0.05	< 0.05
Mean ABS, %	CHX + ADS + HA	NS	NS	NS
	CHX + ADS	NS	NS	NS
	CTRL	NS	NS	NS
Mean Plaque, %	CHX + ADS + HA	NS	NS	NS
	CHX + ADS	NS	NS	NS
	CTRL	NS	< 0.05	NS

*ABS* angulated bleeding score, *ADS* anti-discoloration system, *CHX* chlorhexidine, *CTRL* control, *EHI early healing index*, *HA* hyaluronic acid, *NS* non-significant

## Discussion

In our study, the post-operative usage of chlorhexidine with anti-discoloration system (ADS) was associated with improved wound healing and reduction in both plaque accumulation and marginal inflammation. In addition, the adjunct of hyaluronic acid to this compound determined early wound healing enhancement as measured three days after surgery.

The use of chlorhexidine is well-established in the control of supragingival plaque formation after periodontal surgery [10]. The broad-spectrum antimicrobial activity of chlorhexidine prevents biofilm formation and reduces gingival inflammation, and both effects are fundamental especially during the first post-operative days, due to the suspension/alteration of mechanical plaque removal [19, 24, 28, 36, 38, 46]. Indeed, our control group clearly indicated that in the immediate post-operative period plaque accumulates even in well-trained patients such as the ones that are eligible to the surgical intervention. A concentration of 0.2% chlorhexidine is often considered the gold standard in post-operative care due to its effectiveness in reducing plaque formation by 30–80% [6]. Indeed, less inflammation when compared to control has been noted in the first post-operative week with the administration of post-operative chlorhexidine [13].

However, the usage of post-operative chlorhexidine has been limited by the fact that it is not exempt from some side effects, such as tooth/tongue staining and alterations of taste. Moreover, a delaying effect on wound healing has also been hypothesized. Therefore, the post-operative clinical applications have often been limited. To overcome these side effects, ADS in adjunct to chlorhexidine has been reported to reduce staining, taste alteration and salt perception, without affecting chlorhexidine antimicrobial activity and effectiveness in terms of gingival inflammation and plaque scores [45]. Cortellini and co-workers indicated that the adjunct of ADS did not determine inferior results than chlorhexidine alone, yet showing to reduce pigmentation, cause less side effects and be more acceptable for patients after periodontal surgery [13]. These findings have also been confirmed by Trombelli and co-workers, indicating that chlorhexidine usage, either with ADS and hyaluronic acid or alone, could provide adequate plaque control and decrease gingival inflammation, while guaranteeing optimal wound healing during the first three post-operative weeks [42]. Accordingly, our test group rinsing with chlorhexidine and ADS showed higher plaque control and reduced gingival inflammation when compared to the control group.

Previous in vitro evidence suggested a possible cytotoxic effects of chlorhexidine on some cell types, including gingival fibroblasts, and therefore a possible delay in wound healing was hypothesized [20]. Thus, the immediate post-surgical application of chlorhexidine has been discussed [12]. Nevertheless, our findings do not support this hypothesis as early healing, measured at the third post-operative day, indicated that the adjunct of chlorhexidine-ADS, as seen in both experimental groups, did not produce inferior wound healing if compared to the control group. In fact, improved flap closure was noticed at three days in the interdental papilla and, in the group with hyaluronic acid, even in the experimental incision. These findings altogether would in fact corroborate the idea that plaque control is essential in modulating oral wound healing.

In our study a group with chlorhexidine and ADS was also added with hyaluronic acid. It is worth of attention that over the two weeks of observation, the adjunct of hyaluronic acid significantly yielded the lowest Early Healing Index scores, i.e. higher healing, both at the level of the experimental incision and the interdental papilla versus control group. The rationale of higher wound healing may be explained with the several biological functions exerted by this glycosaminoglycan. Indeed, regulation in cell adhesion, proliferation and differentiation, and in mediating cellular signaling has been reported [5, 17]. Hyaluronic acid has a relevant role in modulating wound healing process, especially in the early stages of cell migration and subsequent differentiation [16, 31]. Moreover, hyaluronic acid appears to enhance early cutaneous wound healing and its concentration rapidly increases at the level of the wound site with the contribution of both platelets and damaged endothelial cells, and peaks after three days from wound development [4]. It may be speculated that this effect may be noted in the oral mucosa as well.

It should be mentioned that in the present study both the formulations of chlorhexidine contained anti-discoloration system. Anti-discoloration system has been reported to improve the control of gingival inflammation, presumably due to the presence of ascorbic acid contributing to the reduction in gingival bleeding tendency [21]. Conversely, an adjunctive effect on plaque reduction was not observed, although the induction of a qualitative alteration of dental plaque has been hypothesized [3]. The reduction in gingival inflammation and bleeding score appeared more evident in the group treated with chlorhexidine and hyaluronic acid at 3 days follow-up, and both the chlorhexidine-based formulations showed beneficial effect on bleeding score and plaque reduction when compared with the control group at days 7 and 14.

The authors are aware of the limitations of the current study. Firstly, the compliance of the participants in the test groups was not formally assessed apart from the collection of the mouth rinse bottles. However, all the included patients presented to all the follow-up visits and completed the study, and it thus can be assumed that the patients were highly motivated. The post-operative evaluation period was limited to the first 14 days, although complete healing may be obtained after 42 days or more. The dynamics of early wound healing could have also been evaluated by administering the two chlorhexidine formulations after the first 24–48 h post-operatively. Another limitation is the lack of placebo in the control group. However, literature has clearly indicated that the usage of control sham mouth rinse such as saline solutions might limit compliance as subjects are probably conscious of not receiving active treatment and this hampers adherence to the study [26]. Suturing the releasing incision only in some patients hindered full standardization of the procedure, however, we deemed necessary to achieve primary stability of the flap, which in some cases requested additional support. Finally, evaluation on larger study samples is needed to further corroborate the present results. Nevertheless, all the examiners reached a satisfactory Kappa score before they started collecting clinical data.

In conclusion, both CHX + HA and CHX were more effective than negative control in enhancing the healing process of periodontal surgical wounds, particularly in the first post-operative week. Interestingly, in the early healing days, the addition of HA appears to enhance primary closure. Further studies to confirm these findings and appraising the potential role that hyaluronic acid could exert on cell migration and differentiation during the early healing period are needed.

### Electronic supplementary material

Below is the link to the electronic supplementary material.

Supplementary file1 (TIFF 3217 KB)

Supplementary file2 (TIFF 3195 KB)

## Figures and Tables

**Fig. 3:**
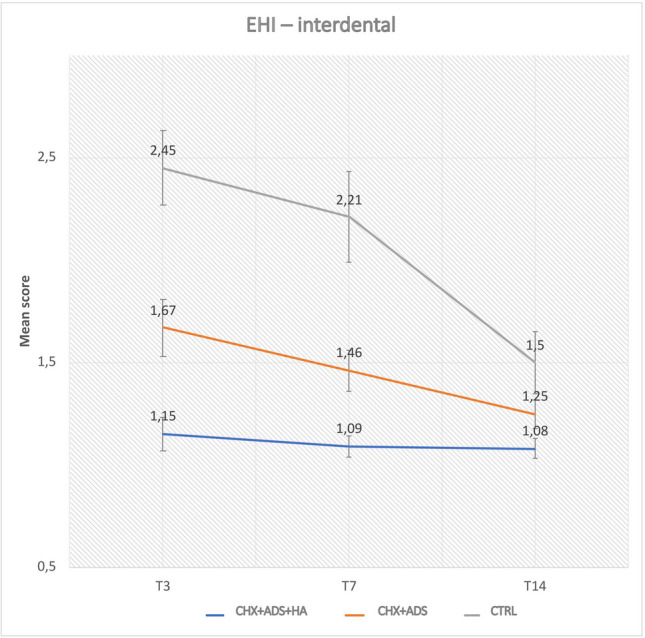
Variation between timepoints of the Early Healing Index (EHI) assessed at the interdental papilla

**Fig. 4:**
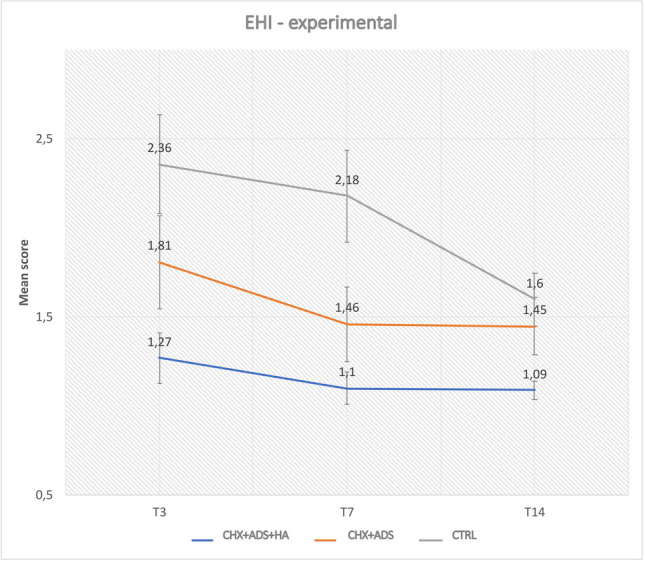
Variation between timepoints of the Early Healing Index (EHI) assessed at the experimental incision

## Data Availability

No datasets were generated or analysed during the current study.

## Appendix 1. Graphical representation of EHI scores at the study timepoints

Figures 3 and 4
