# Supplementary figures and images for: Early periodontal wound healing after chlorhexidine rinsing: a randomized clinical trial
Source: Clin Oral Investig. 2024 Jun 4;28(6):354. doi: 10.1007/s00784-024-05643-0 (PMC11150287; doi:10.1007/s00784-024-05643-0)

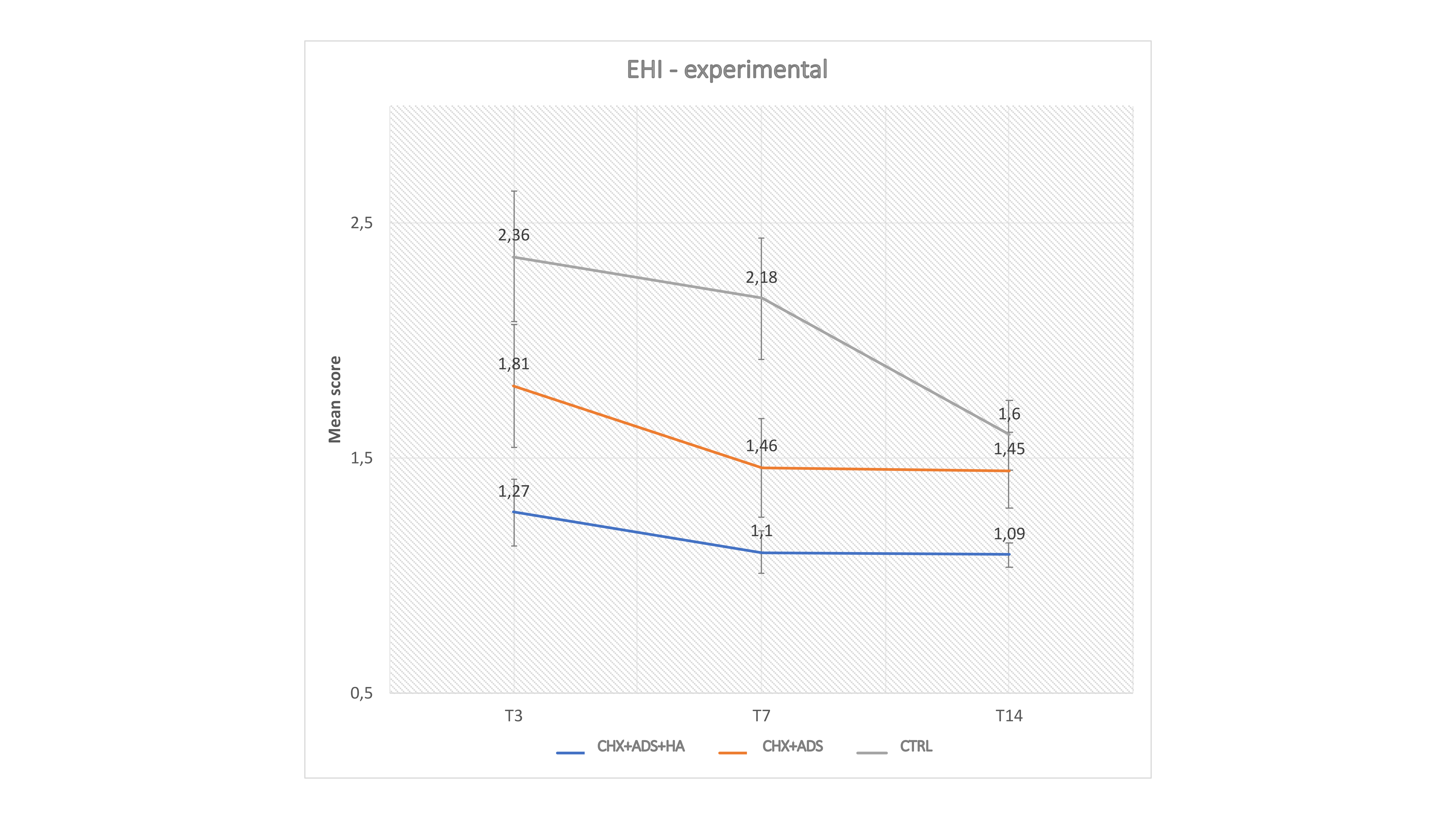

Supplement: Supplementary file 1 — Supplementary file1 (TIFF 3217 KB) [file 784_2024_5643_MOESM1_ESM.tiff]

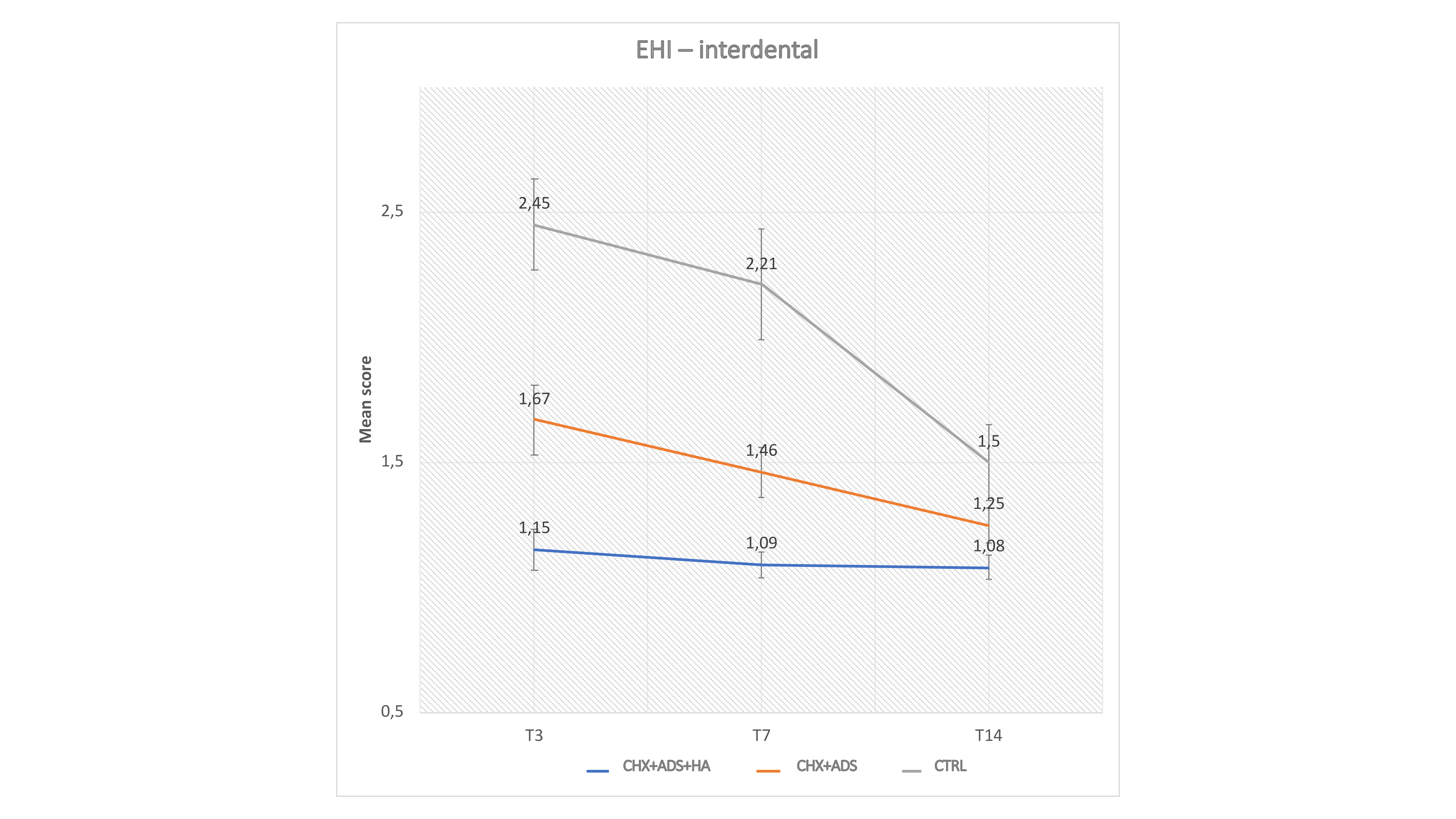

Supplement: Supplementary file 2 — Supplementary file2 (TIFF 3195 KB) [file 784_2024_5643_MOESM2_ESM.tiff]
